# Supplementary material for: On the potential of vehicle-to-grid and second-life batteries to provide energy and material security
Source: Nat Commun. 2024 May 16;15:4179. doi: 10.1038/s41467-024-48554-0 (PMC11099178; doi:10.1038/s41467-024-48554-0)
Supplement: Supplementary file 1 — Supplementary Information [file 41467_2024_48554_MOESM1_ESM.pdf]

Supplementary information for the article: On the potential of vehicle-to-grid and second-life batteries to provide energy and material security

Author list

Fernando Aguilar Lopez[*1], Dirk Lauinger[2,3], François Vuille[2], and Daniel B. Müller[1]

Affiliations

[1] Norwegian University of Science and Technology, Trondheim, Norway

[2] Ecole polytechnique fédérale de Lausanne, Lausanne, Switzerland

[3] Massachusetts Institute of Technology, Cambridge, USA

*fernando@siempreenergy.com

1. Supplementary information to the methods

The overarching goal of our study is to estimate the raw material implications of substituting batteries built specifically for grid storage by vehicle-to-grid and second-life batteries. Our estimates rely on a material flow analysis that calculates battery material needs based on the evolution of the passenger vehicle fleet and of grid storage in the EU. In its most basic form, material flow analysis relies on the principle of mass conservation to track the flows and stocks of chemical elements in spatially-defined balance volumes, so-called *processes*[1–3]. The flows and stocks of chemical elements in battery materials depend on the flows and stocks of batteries, which in turn depend on the flows and stocks of cars. We extend the principle of mass conservation to cars and batteries by carefully accounting for creation and destruction[4,5]. This method has previously been used to model the stock of lithium-ion batteries in electric vehicle fleets[6].

In our model, the demand for passenger vehicles can be met by different vehicle types, *e.g.*, battery electric vehicles, plug-in hybrid electric vehicles, and internal combustion engine vehicles. Similarly, the demand for grid storage can be met by different battery types, *e.g.*, vehicle-to-grid, second-life batteries, and new stationary batteries. While we treat the composition of the vehicle fleet as a fixed input parameter, the composition of the battery fleet for grid storage is a model output that depends on a predefined hierarchy for choosing between battery types.

1.1 Vehicle stock calculations

The demand for vehicles is assumed to be driven by the population and the vehicle ownership per capita as shown in Figure 4 in the main manuscript. The stock can then be calculated as the product between

the population and vehicle ownership per capita. For this model, we relied on the baseline scenario from the United Nations[7] for the EU population projections until 2050 and on the International Organization of Motor Vehicle Manufacturers[8] for the vehicle stock of the same region. Based on the historical values of population and vehicle stock, we calculate the historical vehicles per capita and perform a projection until 2050 based on those values. The resulting vehicles per capita scenarios are multiplied by the population to obtain the vehicle stock. Figure 1 shows the values assumed for each parameter.

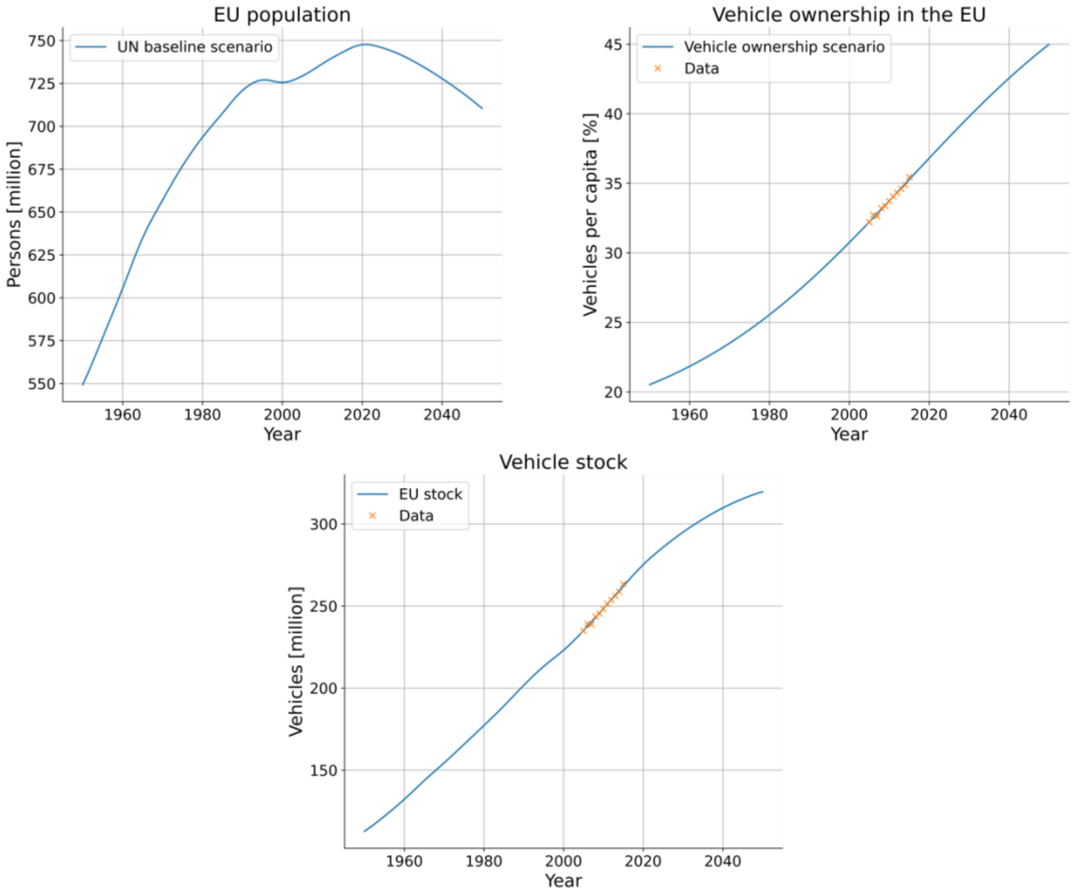

Supplementary Figure 1: Vehicle stock scenario and model drivers for its calculation.

1.2 EV battery lifetime and degradation

We assume that the lifetime of all vehicles follows a normal distribution with a mean of 15 years and a standard deviation of 5 years, which corresponds to the current lifetime of internal combustion engine vehicles (ICEVs) in the EU[9]. Initially, EVs were thought to have a shorter lifetime than ICEVs due to battery degradation but recent experience suggests these concerns were exaggerated[10–12]. In addition, we neglect the impact of vehicle-to-grid on battery lifetime. This may seem counterintuitive because V2G leads to an increased energy throughput, which is generally known to accelerate battery ageing[13]. However, V2G also leads to slower charging rates and a lower average state-of-charge, which improve battery health and may more than compensate for the increased energy throughput[14,15]. In fact, when traditional EVs are connected to charging stations, they are often charged as fast as possible. With V2G,

the charging rate is modulated by the storage needs of the electricity grid. If the grid needs power at a particular moment, then EVs will charge below their desired charging rates or even discharge power back into the grid. If there is too much power in the grid, then EVs will charge above their desired charging rates. For EVs to be able to adjust their charging rates in this manner, the desired charging rates must be set below the maximum capacity of the vehicle charger and hence below the rates of traditional charging. On average, EVs may be providing and consuming similar amounts of power to and from the grid for V2G but they often do not know in advance when they will be called upon to provide power and when they will be called upon to consume power. It is thus usually optimal to maintain a state-of-charge well below 100% to maximize the amount of storage power that can be sold to the grid. For frequency regulation, which is often regarded as one of the most profitable applications of vehicle-to-grid, it is optimal to maintain a state-of-charge of about 50%, for example[16]. Finally, the amount of storage made available to the grid is often chosen in such a way that the battery experiences only modest deviations in its state-of-charge[17,18]. Peak shaving, which consists of charging when electricity consumption is low and discharging when electricity consumption is high, is another application that may lead to negligible or even negative additional battery degradation[19,20]. However, the long-term battery degradation induced by vehicle-to-grid has not been tested extensively yet. It is thus conceivable that there may be applications for which vehicle-to-grid will shorten battery life. In this study, we assumed that vehicle-to-grid does not shorten battery life given the lack of evidence for the impact of V2G on battery degradation.

We assume that EV batteries have around 80% of their initial capacity left when they reach the end of their automotive life[21,22]. We consider that batteries older than 15 years degrade faster than younger batteries and thus consider a concave piecewise linear degradation curve. As can be seen in Figure 2, in our model most vehicles (68%) reach their end of life when they are between 10 and 20 years old. The remaining vehicles have a longer or shorter in use time, which accounts for differences in lifestyle choices and use habits. About 2.5% of all vehicles remain in use for less than 5 years, which accounts for accidents, battery failures, and other causes for early obsolescence. While it is true that batteries and EVs could have different lifetimes that result in different causes for obsolescence, we calculate the system dynamics using only one distribution to reduce the sensitivity of the model to specific assumptions and maximize interpretability.

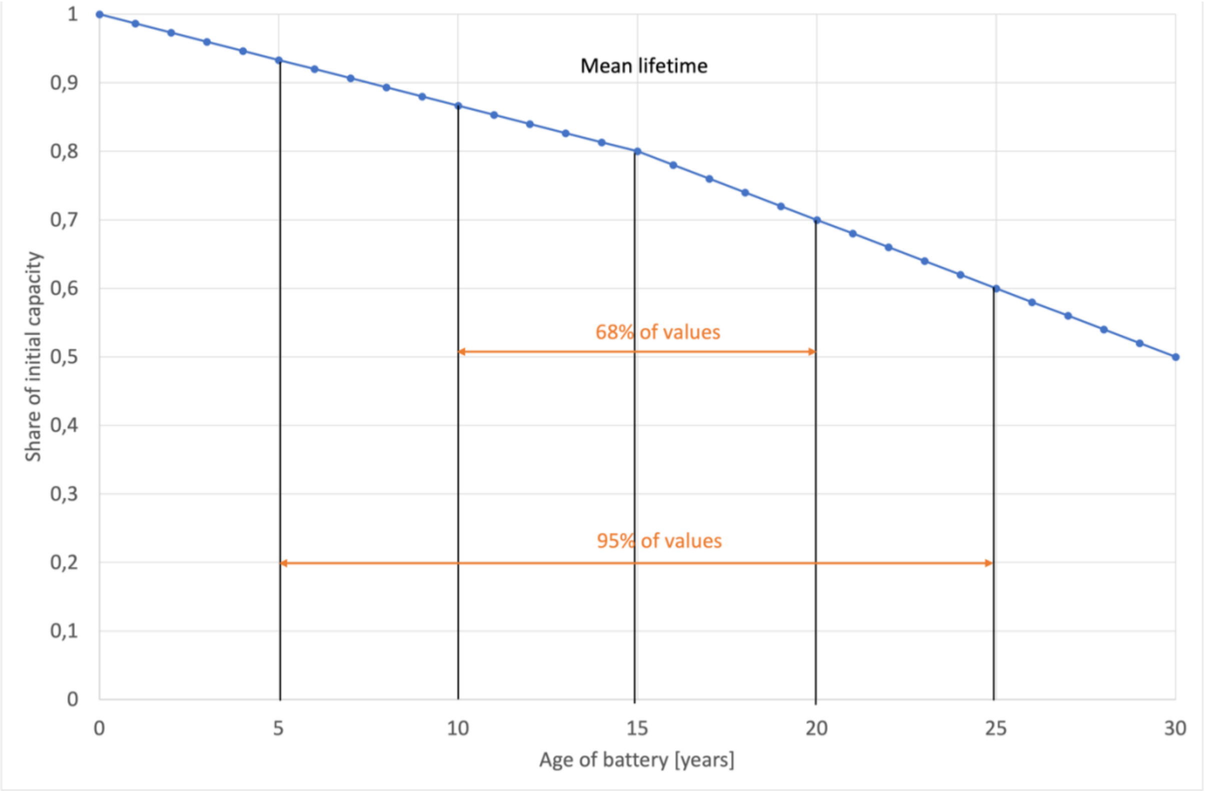

Supplementary Figure 2: Degradation curve for batteries in electric vehicles.

1.3 Battery reuse, lifetime, and degradation

We consider three scenarios for the reuse rate of end-of-life EV batteries: no reuse, reuse of LFP batteries, and reuse of all batteries. In the LFP- and all-reuse scenarios, we consider that end-of-life EV batteries enter the second-life stock with 80% of their initial capacity. Given the lack of second-life batteries with current EV battery chemistries, there is little evidence on which to base assumptions about the lifetime of future second-life batteries[23]. Here, we assume a lifetime of 6 years with a standard deviation of 2 years in accordance with the scant literature on the topic[21]. For new batteries, we assume a mean lifetime of 20 years with a standard deviation of 4 years. We consider linear degradation curves for both second-life batteries and new stationary batteries. Thus, the total lifetime of the batteries remains at 20 to 21 years on average regardless of their use. However, to account for the higher variability in user-specific battery degradation, EV batteries can leave the stock significantly earlier than stationary batteries and not be reused, resulting in a larger standard deviation for their obsolescence.

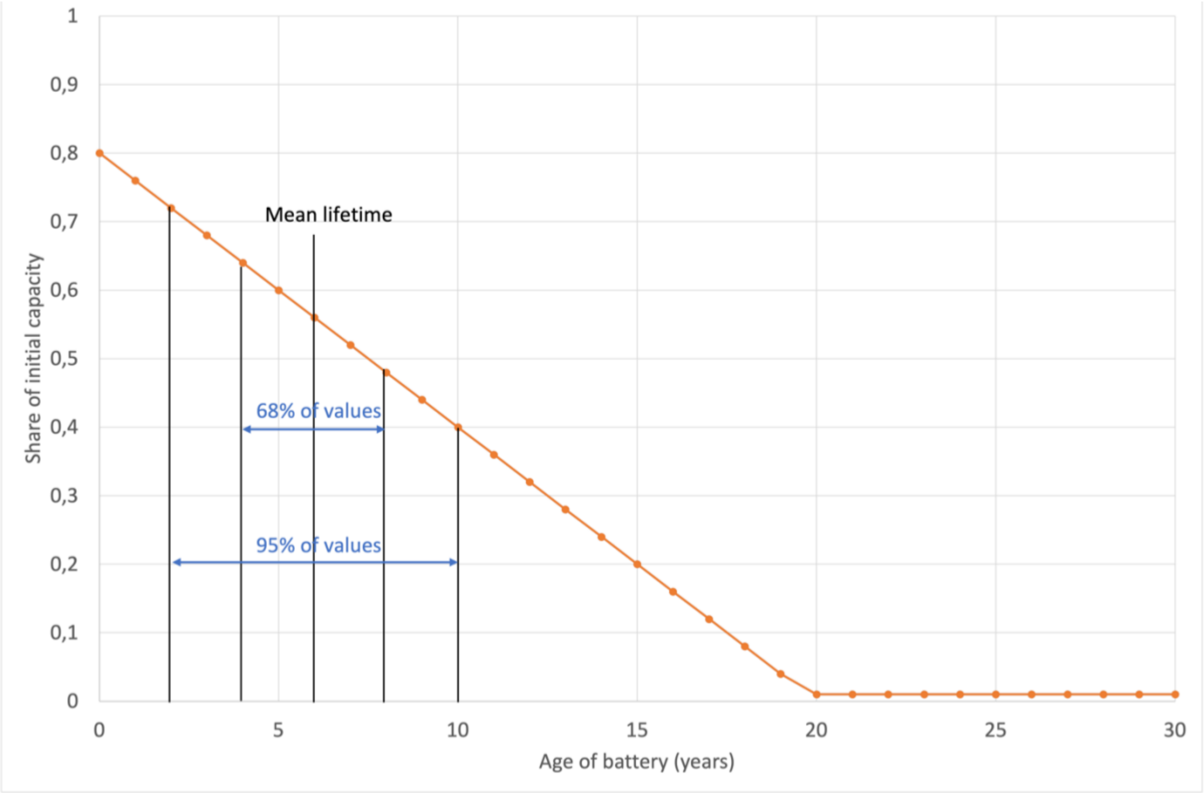

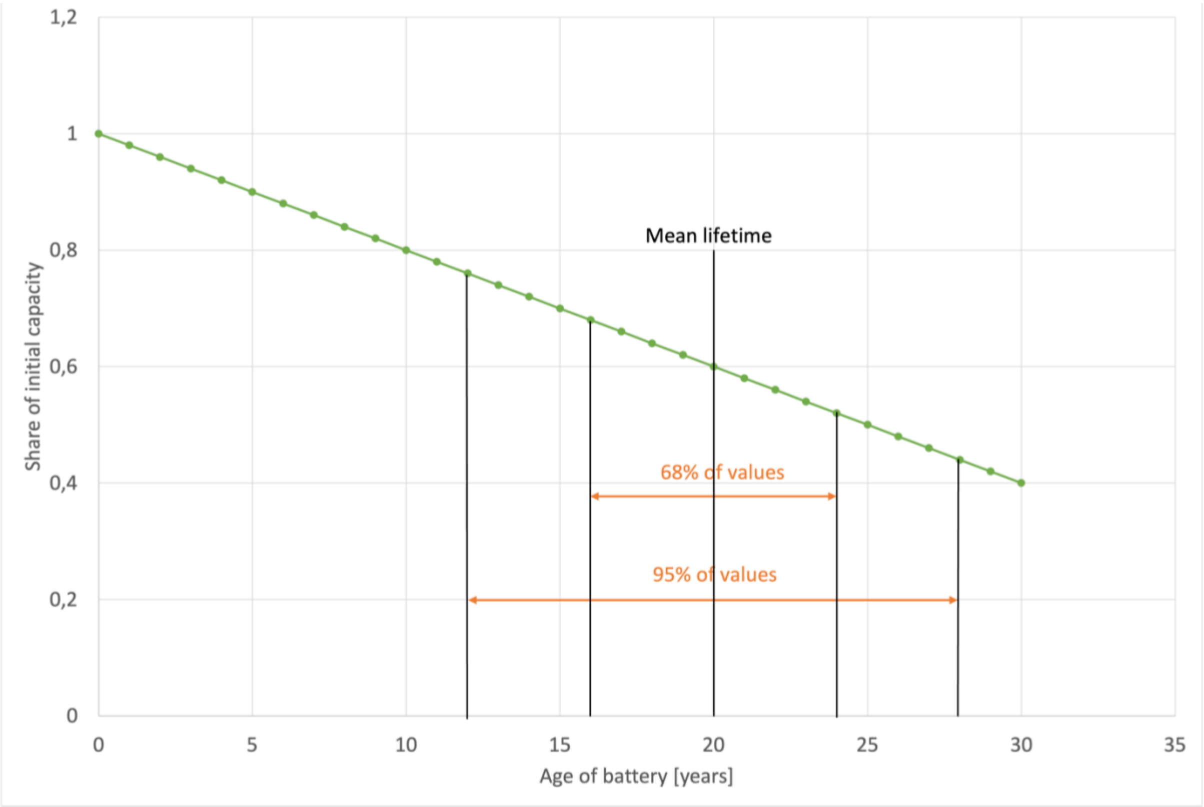

Supplementary Figure 3: Degradation curves for second-life batteries and new stationary batteries,

1.4 Electric vehicle penetration scenarios

The penetration of BEVs and PHEVs is calculated as a share of total vehicle sales and based on the Stated Policies scenario, the Announced Pledges scenario, and the Net Zero Emissions by 2050 scenario

from the International Energy Agency (IEA)[24]. Our scenarios are also informed by reports from the European Commission (EC)[25] and by the European Network of Transmission System Operators for Electricity (ENTSOE)[26,27], which publish scenarios on the share of electric vehicles in the overall vehicle fleet as opposed to in vehicle sales. The Net Zero Emissions by 2050 scenario has a fuel cell penetration of 10% by 2050. The EC and the ENTSOE compute with a larger range, they estimate that about 5-20% of the vehicle fleet will be fuel cell electric by 2050. In this study, we define two scenarios: the baseline scenario follows the lower end of the IEA penetration scenarios for BEVs and results in a 10% fuel cell sale penetration by 2050, the accelerated scenario follows the upper end of the IEA penetration scenarios for BEVs and results in a 20% fuel cell sale penetration by 2050.

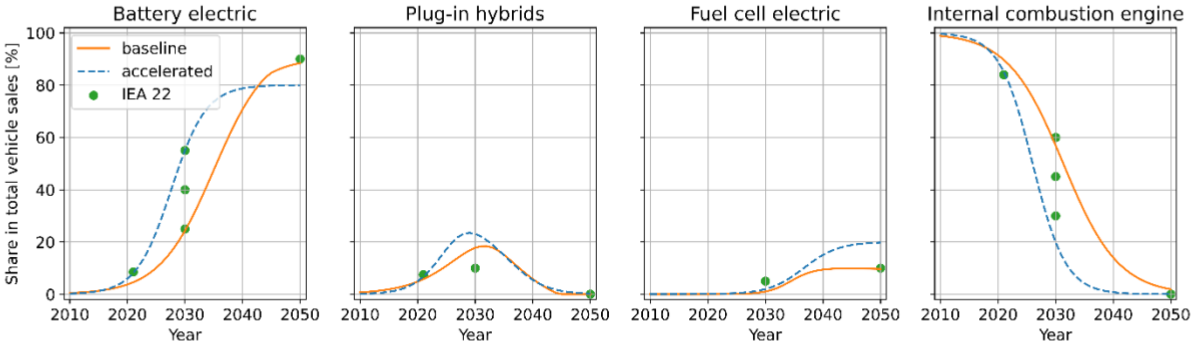

Supplementary Figure 4: EV sales penetration scenarios.

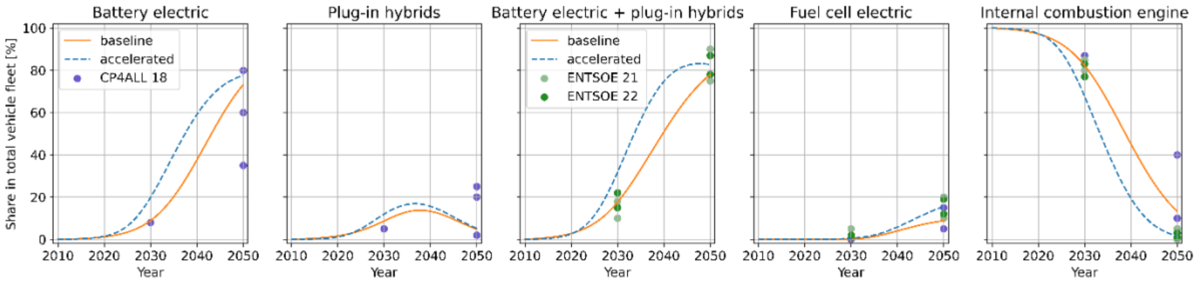

Supplementary Figure 5: EV fleet penetration scenarios.

1.5 Vehicle and battery size scenarios

The vehicle and battery sizes were adapted as proposed by Xu et al.[28], with constant shares over the entire period. BEVs can have small (33 kWh), medium (66 kWh), or large (100 kWh) batteries, while PHEVs have smaller batteries of 8 kWh, 12 kWh, and 17 kWh, respectively. We assume that all BEVs are capable of fully charging overnight, in 6 hours, and that bidirectional charging stations will be sized such that BEVs could fully discharge in the same amount of time. The ratio of charging power and battery size thus matches the requirements for grid storage discussed in section 1.9.

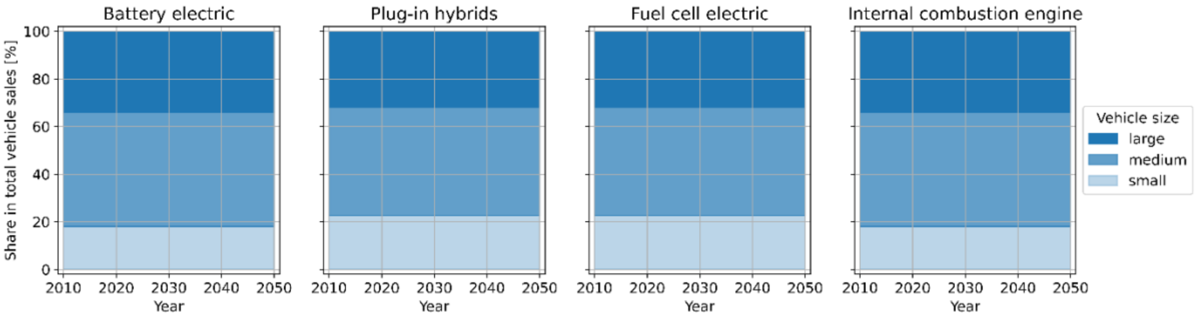

Supplementary Figure 6: Vehicle size scenarios.

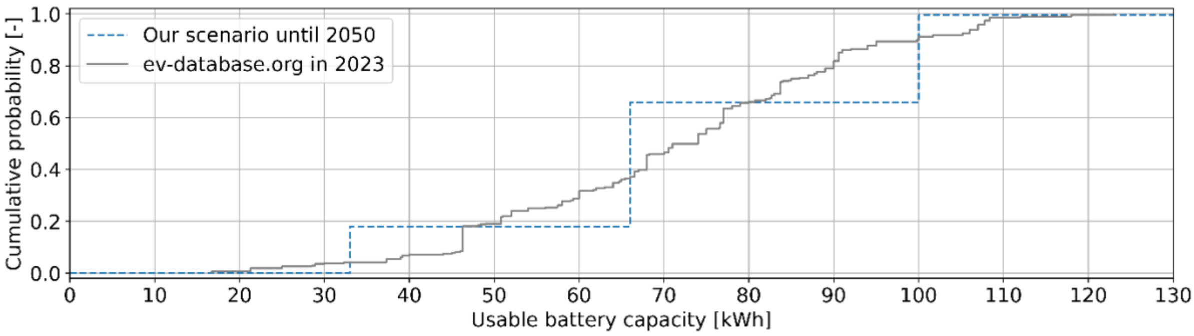

Supplementary Figure 7: Usable battery capacity among vehicle models.

1.6 Vehicle-to-grid penetration scenarios

We consider four different scenarios (none, low, medium, and mandate) for the share of V2G in BEV sales. On an average day, over 90% of all cars are parked at any one time[29,30]. In order to compute the available storage capacity from V2G, we further distinguish whether vehicles that are equipped with V2G are connected to bidirectional charging stations. In general, vehicle owners may only connect their cars to charging stations if they are concerned about their battery state-of-charge being low. The ongoing increase in battery capacity of new EV models and in charging speeds of new EV chargers may mean that vehicle owners will connect less often to charging stations when they park. In this study, we assume that vehicle owners who invested in V2G will connect to charging stations more regularly to take advantage of the revenue from providing storage to the grid. Specifically, we assume that 50% of all cars equipped with V2G will be connected to the grid at any one time, which means that parked cars with V2G are connected to the grid 55.6% of the time, and that, on average, each of them makes half of its battery capacity available for V2G. It may seem unrealistic that vehicle owners set apart half of their battery capacity for V2G. However, the average daily driving distance in the EU is about 30km[31] and already today virtually all new EVs in the EU have a range greater than 150km (see Figure 6, derived from the "*EV database*", accessible online at: *ev-database.org*), EV drivers are thus still able to cover 2.5 times their average daily driving distance if they set apart 50% of their battery capacity for V2G. Given their smaller battery size, we assume that PHEVs do not participate in V2G. We assume the same V2G penetration rate for BEVs of all sizes regardless of their battery chemistry.

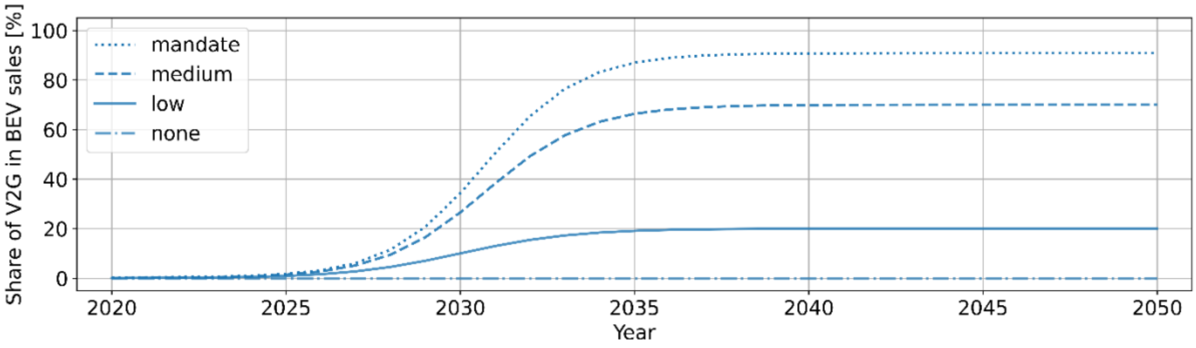

Supplementary Figure 8: V2G penetration scenarios.

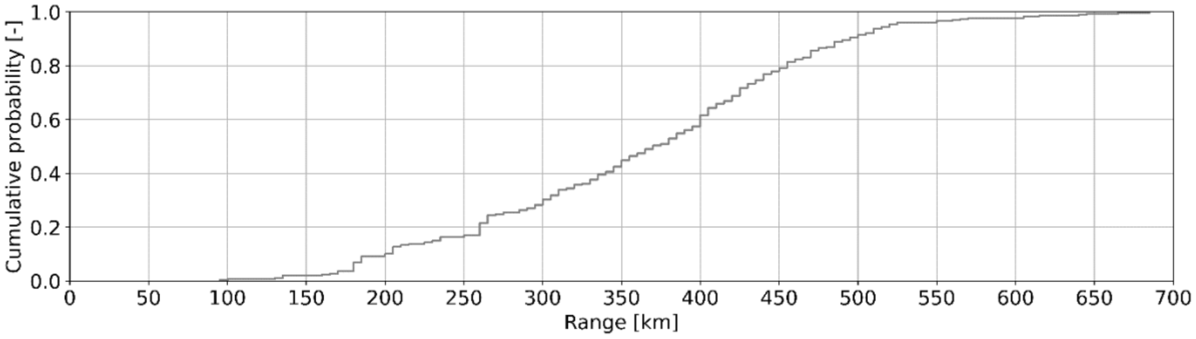

Supplementary Figure 9: Range of commercially available EVs in the EU in 2023.

1.7 Battery chemistry development

For the split of battery chemistries in new EVs, we follow a baseline scenario defined by Bloomberg New Energy Finance[32] until 2030 and keep them constant thereafter as suggested by Aguilar Lopez et al.[10]. When calculating the raw material needs for battery production, we aggregate over the battery materials (Li, Co, Ni, P, Mn, C) to reduce the sensitivity of our results to future changes in battery chemistries. Furthermore, we only consider the materials in the battery modules to reduce the uncertainty related to the role of battery dismantling and reuse in second life, as these materials would not be spatially separated.

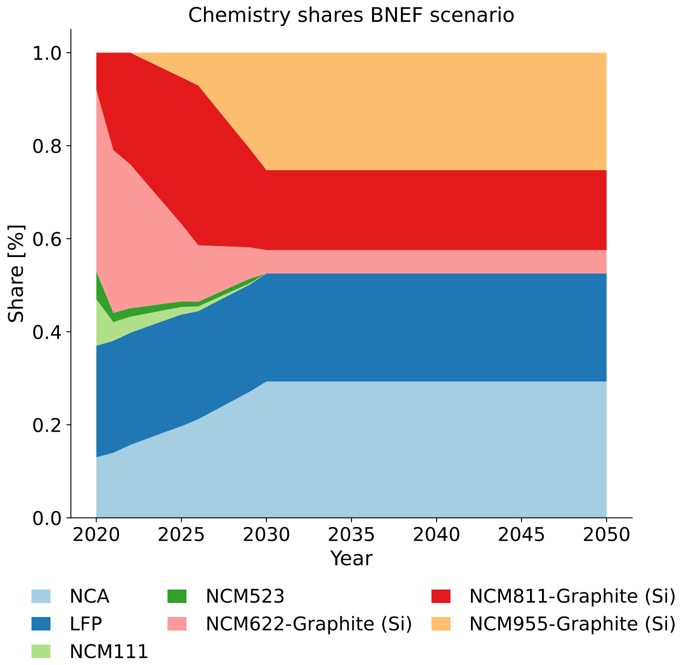

Supplementary Figure 10: Battery chemistry development until 2050.

1.8 Recycling efficiencies

We define the material-specific recycling efficiencies according to the three main recycling technologies: pyrometallurgical, hydrometallurgical, and direct recycling. The values are taken from literature as introduced by Aguilar Lopez et al. (2023)[10] and summarized in the table below:

Recycling technology	Lithium	Cobalt	Nickel	Phosphorus	Manganese	Graphite
Pyrometallurgical	0%	75%	75%	0%	0%	0%
Hydrometallurgical	40%	80%	80%	0%	50%	40%
Direct recycling	90%	90%	90%	90%	90%	90%

1.9 Stationary storage demand scenarios

The demand for battery storage for the electricity grid is based on reports by the EC and by the ENTSOE. In its 2020 study on energy storage and in the impact assessment accompanying the communication on *"Stepping up Europe's 2030 climate ambition"*, the EC estimates battery storage

needs between 20-40GW by the year 2030 and between 50-120GW by the year 2050[33,34]. In its *"Clean Planet for all"* communication from 2018, the EC projected battery storage needs of up to 180GW by 2050[25]. In 2021, before the war in Ukraine and the shutdown of over half the French nuclear power plants, the ENTSOE calculated with a narrower range of 10-40GW by 2030, 75-100GW by 2040 and 90-100GW by 2050 in the draft for its 2022 ten year network development plan[26]. In 2022, the ENTSOE increased its estimates to 7GW by 2025, 20-100GW by 2030, 40-170GW by 2040, and 140-240GW by 2050[27]. In this study, we define three scenarios: the low scenario follows the lower end of the 2020 estimates by the EC, the medium scenario follows the medium projections by the EC and the 2021 projections by the ENTSOE, and the high scenario follows the higher end of the 2022 projections by the ENTSOE. Both the EC and the ENTSOE estimate battery storage needs in terms of power. We assume that battery owners will have to be able to provide power for 6 hours a day. The battery storage needs in Figure 1 of the main paper are thus computed by multiplying the storage needs in terms of power by 6h. This assumption is in line with EC market regulations for primary frequency regulation[35]. We also assume that electric vehicles providing V2G are parked at charging stations that are powerful enough to fully charge or discharge the amount of energy that electric vehicles make available for V2G, half of the battery capacity on average, within 6 hours. Similarly, we assume that all stationary batteries can fully charge or discharge within 6 hours.

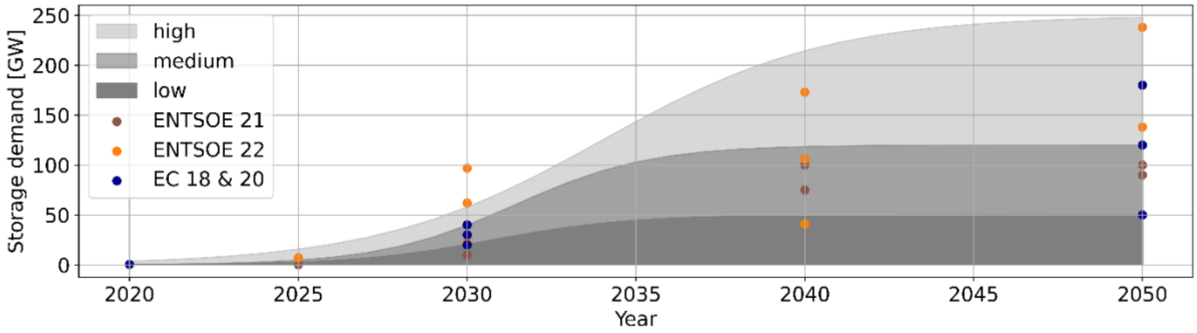

Supplementary Figure 11: Storage demand scenarios.

1.10 Model description

The unconstrained model works using the parameters described above in a traditional material flow analysis way[2,4,36] based on the mass balance principle. Consider for example the vehicle stock (Process 3 in Figure 4 in the main manuscript). We compute the size of the vehicle stock v_t at the end of any given year t in our planning horizon as the product of two exogenous parameters: the population and the number of vehicles per capita. Next, we will determine the number of vehicles v_t^+ that enter the fleet in year t and the number of vehicles v_t^- that are retired from the fleet in year t. The discrete lifetime distribution in Figure 2 specifies the fraction $L_{t\tau} = v_\tau^- / v_t^+$. Given the evolution of the vehicle fleet and the lifetime distribution, we can thus compute the inflows as $\mathbf{v}^+ = \Delta\mathbf{S}\,(\mathbf{I} - \mathbf{L})^{-1}$ and the outflows as $\mathbf{v}^- = \mathbf{v}^+ - \Delta\mathbf{S}$, where $\mathbf{v}^+$ and $\mathbf{v}^-$ are row vectors of the in- and outflows in all years, respectively, $\Delta\mathbf{S}$

is a row vector of stock changes given by the first difference of the vehicle fleet, **I** is the identity matrix, and **L** is a lifetime matrix constructed such that $L_{t\tau}$ is in row τ and column t. In this model, all EVs that can be equipped with V2G will be equipped with V2G and all EV batteries that can be reused will be reused, regardless of whether there is or will be a demand for additional storage for the electricity grid.

In practice, the installed capacity for grid services should not exceed the demand as there may be no market for it. We thus performed a demand-constrained analysis to investigate the emerging competition between V2G, SLBs, and NSBs in satisfying the need for stationary storage. We assumed in particular that the newly installed storage capacity in any year exactly matches the demand for new capacity in that year. We prioritized the installation of the different storage technologies as follows:

1) The available V2G capacity is installed.
2) If the storage demand still exceeds the installed capacity, the available SLBs are installed.
3) If there is still a gap between the installed capacity and demand, this gap is exactly matched by installing NSBs.

If the available capacity of V2G or SLB storage surpasses the demand for new storage, only a fraction of that capacity is installed. The surplus of potentially available storage is not installed. Any excess electric vehicles are not equipped with V2G and any excess SLBs are directly collected for recycling.

All details about the numerical implementation can be found in the code, which is made fully available with an open license to ensure reproducibility.

2. Ratio of installed to potential capacity for all V2G and reuse scenarios

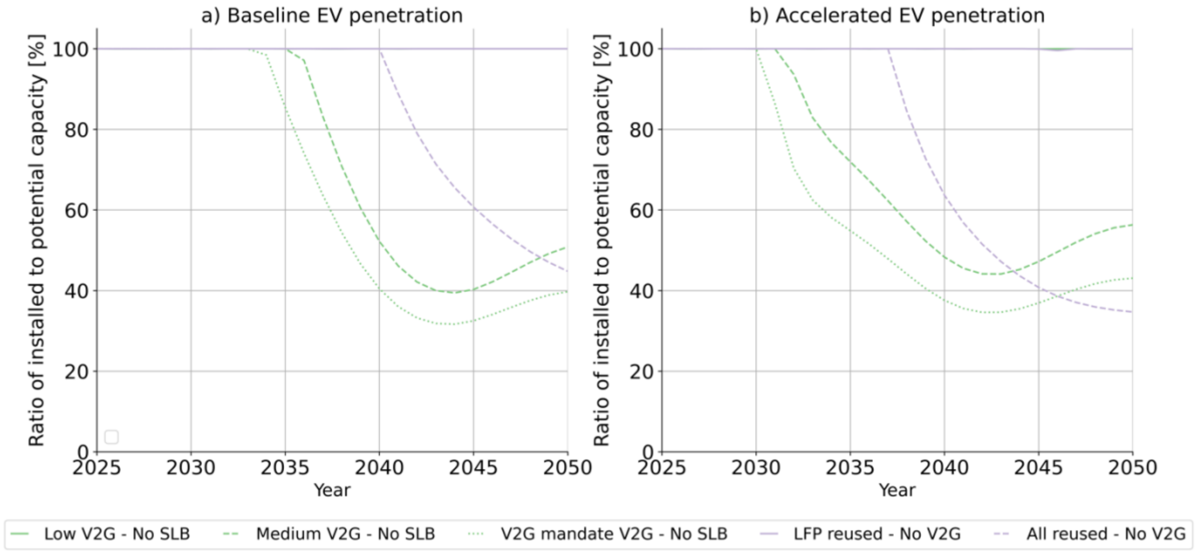

Supplementary Figure 12: Ratio of installed to potential capacity (utilization factor) for V2G and SLBs in the high demand scenario under a) the baseline EV penetration and b) the accelerated EV penetration.

3. Disaggregated material demand

The following figure shows the disaggregated values presented in Figure 2 in the main manuscript to show the material demand by element.

Since the demand for stationary storage experiences its sharpest increase between 2030 and 2040, this growth period is decisive for the different technologies to establish themselves in the market. Our V2G mandate scenario assumes a 35% V2G penetration rate in vehicles sales by 2030, leaving some lead time for the regulatory and behavioral changes needed for the large-scale adoption of V2G. Once these barriers are surpassed, we assume a sharp increase to 90% by 2035. As the increase of V2G in EV sales is well aligned with the increase in stationary storage demand, the electrification rate of the vehicle fleet has only a limited influence on the penetration of V2G in the stationary storage market (see Figures 13b and 13h). The vehicle electrification rate is more relevant for SLBs, which become available for grid storage only after their automotive life. A slow EV adoption delays the availability of SLBs in large volumes, which results in SLBs not being able to cover the demand for new storage during the growth period. More NSBs are thus installed to complement SLBs (see Figures 13c and 13i). Overall, V2G has a greater potential than SLBs to reduce the need for NSBs and thus for primary materials during the critical phase of 2030-2040 when the demand for storage increases sharply.

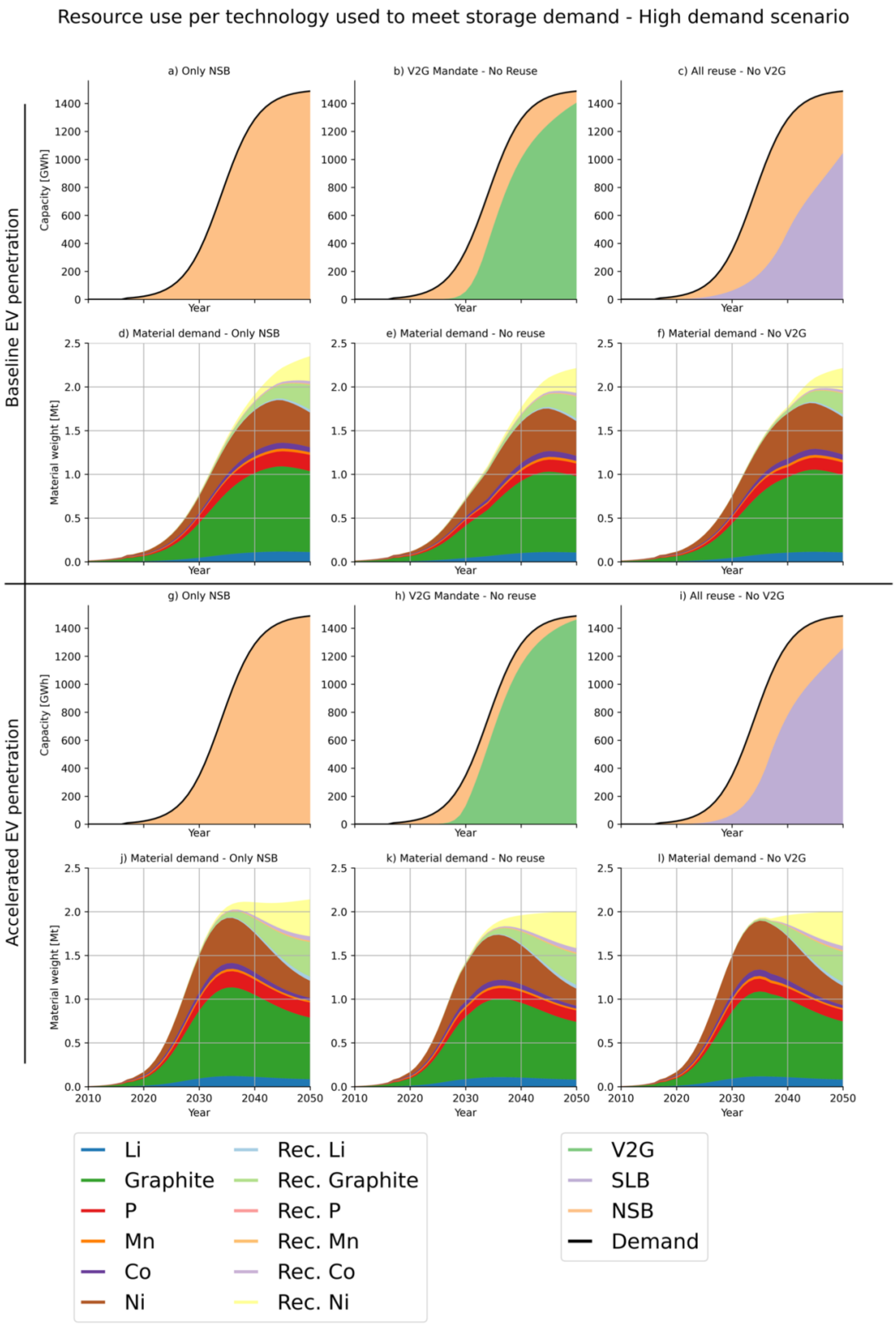

Supplementary Figure 13: Disaggregated material demand corresponding to Figure 2 in main manusctript. Graphs a and g show the capacity of only using NSBs and d and j show the corresponding disaggregated material demand. Figures b, e, h, and k show the corresponding graphs for the high V2G penetration scenario without reuse and c, f, i, and l show the same values for the high reuse scenario without V2G.

4. Material demand under pyrometallurgical, hydrometallurgical, and direct recycling

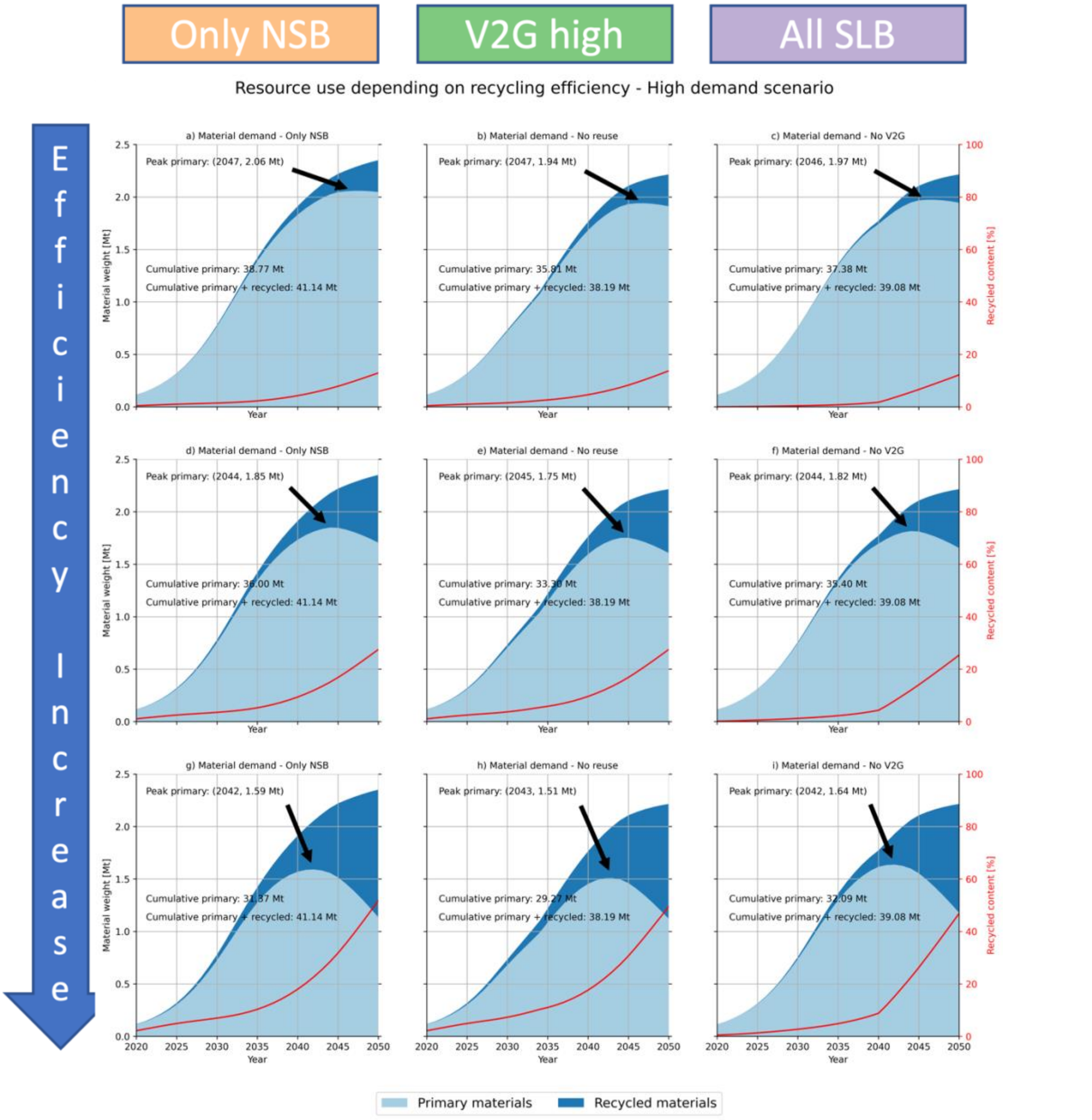

Supplementary Figure 14: Dependence of primary material demand and reuse on recycling efficiency. (Top row (a, b, c): pyrometallurgical recycling, middle row (d, e, f): hydrometallurgical recycling (g, h, i), bottom row: direct recycling).

Supplementary References

1. Hendriks, C. *et al.* Material Flow Analysis: A tool to support environmental policy decision making. Case-studies on the city of Vienna and the Swiss lowlands. *Local Environment* **5**, 311–328 (2000).
2. Brunner, P. H. & Rechberger, H. *Practical Handbook of Material Flow Analysis*. (Lewis Publishers, Boca Raton, London, New York, Washington D.C., 2004).
3. Baccini, P. & Brunner, P. H. *Metabolism of the Anthroposphere: Analysis, Evaluation and Design*. (MIT Press, Cambridge, Massachusetts; London, England, 2012).
4. Lauinger, D., Billy, R. G., Vásquez, F. & Müller, D. B. A general framework for stock dynamics of populations and built and natural environments. *Journal of Industrial Ecology* **25**, 1136–1146 (2021).
5. Aguilar Lopez, F., Billy, R. G. & Müller, D. B. A product–component framework for modeling stock dynamics and its application for electric vehicles and lithium-ion batteries. *Journal of Industrial Ecology* **26**, 1605–1615 (2022).
6. Thorne, R., Aguilar Lopez, F., Figenbaum, E., Fridstrøm, L. & Müller, D. B. Estimating stocks and flows of electric passenger vehicle batteries in the Norwegian fleet from 2011 to 2030. *Journal of Industrial Ecology* **25**, 1529–1542 (2021).
7. United Nations Department of Economic and Social Affairs. Population Division. *World Population Prospects 2019, Online Edition. Rev. 1.* https://www.un.org/development/desa/pd/news/world-population-prospects-2019-0 (2019).
8. International Organization of Motor Vehicle Manufacturers. *World Vehicles in Use 2015-2020.* https://www.oica.net/category/vehicles-in-use/.
9. Oguchi, M. & Fuse, M. Regional and longitudinal estimation of product lifespan distribution: A case study for automobiles and a simplified estimation method. *Environ. Sci. Technol.* **49**, 1738–1743 (2015).
10. Aguilar Lopez, F., Billy, R. G. & Müller, D. B. Evaluating strategies for managing resource use in lithium-ion batteries for electric vehicles using the global MATILDA model. *RCR* **193**, 106951 (2023).

11. Peters, I. M. *et al.* The role of batteries in meeting the PV terawatt challenge. *Joule* **5**, 1353–1370 (2021).
12. Abdelbaky, M., Peeters, J. R. & Dewulf, W. On the influence of second use, future battery technologies, and battery lifetime on the maximum recycled content of future electric vehicle batteries in Europe. *Waste Management* **125**, 1–9 (2021).
13. Sarre, G., Blanchard, P. & Broussely, M. Aging of lithium-ion batteries. *Journal of Power Sources* **127**, 65–71 (2004).
14. Uddin, K., Dubarry, M. & Glick, M. B. The viability of vehicle-to-grid operations from a battery technology and policy perspective. *Energy Policy* **113**, 342–347 (2018).
15. Lehtola, T. A. & Zahedi, A. Electric Vehicle Battery Cell Cycle Aging in Vehicle to Grid Operations: A Review. *IEEE J. Emerg. Sel. Topics Power Electron.* **9**, 423–437 (2021).
16. Lauinger, D. Vehicle-to-Grid for Reliable Frequency Regulation. (Ecole polytechnique fédérale de Lausanne, 2022).
17. Uddin, K. *et al.* On the possibility of extending the lifetime of lithium-ion batteries through optimal V2G facilitated by an integrated vehicle and smart-grid system. *Energy* **133**, 710–722 (2017).
18. Thompson, A. Economic implications of lithium ion battery degradation for vehicle-to-grid (V2X) services. *Journal of Power Sources* **396**, 691–709 (2018).
19. Bhoir, S., Caliandro, P. & Brivio, C. Impact of V2G service provision on battery life. *Journal of Energy Storage* **44**, 103178 (2021).
20. Hytönen, Eric Johannes. *Vehicle to Grid Battery Degradation Impact*. (2023).
21. Casals, L. C., Amante García, B. & Canal, C. Second life batteries lifespan: Rest of useful life and environmental analysis. *Journal of Environmental Management* **232**, 354–363 (2019).
22. Reinhardt, R., Christodoulou, I., Gassó-Domingo, S. & Amante García, B. Towards sustainable business models for electric vehicle battery second use: A critical review. *Journal of Environmental Management* **245**, 432–446 (2019).
23. Martinez-Laserna, E. *et al.* Battery second life: Hype, hope or reality? A critical review of the state of the art. *Renewable and Sustainable Energy Reviews* **93**, 701–718 (2018).

24. International Energy Agency. *Global EV Outlook 2022 - Securing Supplies for an Electric Future*. https://www.iea.org/reports/global-ev-outlook-2022 (2022).
25. European Commission. *A Clean Planet for All - In-Depth Analysis in Support of the Commission Communication COM(2018) 773*. (2018).
26. European Network of Transmission System Operators for Gas and Electricity. *TYNDP 2022 Draft Scenario Report*. (2021).
27. European Network of Transmission System Operators for Gas and Electricity. *TYNDP 2022 Scenario Report*. https://2022.entsos-tyndp-scenarios.eu/ (2022).
28. Xu, C. *et al.* Future material demand for automotive lithium-based batteries. *Commun Mater* **1**, 99 (2020).
29. Kempton, W. & Letendre, S. E. Electric vehicles as a new power source for electric utilities. *Transportation Research Part D: Transport and Environment* **2**, 157–175 (1997).
30. Shaheen, Susan. Shared Mobility: The Potential of Ridehailing and Pooling. in *Three Revolutions* (ed. Sperling, Daniel) 55–76 (Island Press, 2018).
31. Enerdata. Change in distance travelled by car. *Sectoral Profile - Transport* https://www.odyssee-mure.eu/publications/efficiency-by-sector/transport/distance-travelled-by-car.html (2021).
32. Bloomberg New Energy Finance. *Electric Vehicle Outlook 2021*. https://about.bnef.com/electric-vehicle-outlook/ (2021).
33. Andrey, C. *et al. Study on Energy Storage – Contribution to the Security of the Electricity Supply in Europe*. (Publications Office of the European Union, 2020).
34. European Commission. Stepping up Europe's 2030 climate ambition. (2020).
35. European Commission. Commission Regulation (EU) 2017|1485 of 2 August 2017 establishing a guideline on electricity transmission system operation. *Official Journal of the European Union* **60**, (2017).
36. B. Müller, D. Stock dynamics for forecasting material flows—Case study for housing in The Netherlands. *Ecological Economics* **59**, 142–156 (2006).
