# Supplementary material for: On the potential of vehicle-to-grid and second-life batteries to provide energy and material security
Source: Nat Commun. 2024 May 16;15:4179. doi: 10.1038/s41467-024-48554-0 (PMC11099178; doi:10.1038/s41467-024-48554-0)
Supplement: Supplementary file 3 — Reporting Summary [file 41467_2024_48554_MOESM3_ESM.pdf]

nature portfolio

Corresponding author(s): Fernando Aguilar Lopez

Last updated by author(s): Apr 24, 2024

Reporting Summary

Nature Portfolio wishes to improve the reproducibility of the work that we publish. This form provides structure for consistency and transparency in reporting. For further information on Nature Portfolio policies, see our Editorial Policies and the Editorial Policy Checklist.

Statistics

For all statistical analyses, confirm that the following items are present in the figure legend, table legend, main text, or Methods section.

n/a	Confirmed	
☒	☐	The exact sample size (*n*) for each experimental group/condition, given as a discrete number and unit of measurement
☒	☐	A statement on whether measurements were taken from distinct samples or whether the same sample was measured repeatedly
☒	☐	The statistical test(s) used AND whether they are one- or two-sided<br>*Only common tests should be described solely by name; describe more complex techniques in the Methods section.*
☒	☐	A description of all covariates tested
☒	☐	A description of any assumptions or corrections, such as tests of normality and adjustment for multiple comparisons
☒	☐	A full description of the statistical parameters including central tendency (e.g. means) or other basic estimates (e.g. regression coefficient) AND variation (e.g. standard deviation) or associated estimates of uncertainty (e.g. confidence intervals)
☒	☐	For null hypothesis testing, the test statistic (e.g. *F*, *t*, *r*) with confidence intervals, effect sizes, degrees of freedom and *P* value noted<br>*Give P values as exact values whenever suitable.*
☒	☐	For Bayesian analysis, information on the choice of priors and Markov chain Monte Carlo settings
☒	☐	For hierarchical and complex designs, identification of the appropriate level for tests and full reporting of outcomes
☒	☐	Estimates of effect sizes (e.g. Cohen's *d*, Pearson's *r*), indicating how they were calculated

Our web collection on statistics for biologists contains articles on many of the points above.

Software and code

Policy information about availability of computer code

Data collection	All data were sourced from publicly available repositories and sources as described and duly referenced in the text. The used data is made available in the repository: https://doi.org/10.5281/zenodo.10966035
Data analysis	All data were sourced from publicly available repositories and sources as described and duly referenced in the text. The resulting regressions and forecasts are also made fully available in tabular form in the repository. The Python code used is fully accessible on the following links: github https://github.com/fernaag/V2G_in_EU, zenodo https://doi.org/10.5281/zenodo.10966035

For manuscripts utilizing custom algorithms or software that are central to the research but not yet described in published literature, software must be made available to editors and reviewers. We strongly encourage code deposition in a community repository (e.g. GitHub). See the Nature Portfolio guidelines for submitting code & software for further information.

Data

Policy information about availability of data

All manuscripts must include a data availability statement. This statement should provide the following information, where applicable:

- Accession codes, unique identifiers, or web links for publicly available datasets
- A description of any restrictions on data availability
- For clinical datasets or third party data, please ensure that the statement adheres to our policy

All data used and generated in this study is made fully available in the following repository: zenodo https://doi.org/10.5281/zenodo.10966035

Research involving human participants, their data, or biological material

Policy information about studies with human participants or human data. See also policy information about sex, gender (identity/presentation), and sexual orientation and race, ethnicity and racism.

Reporting on sex and gender	n/a
Reporting on race, ethnicity, or other socially relevant groupings	n/a
Population characteristics	n/a
Recruitment	n/a
Ethics oversight	n/a

Note that full information on the approval of the study protocol must also be provided in the manuscript.

Field-specific reporting

Please select the one below that is the best fit for your research. If you are not sure, read the appropriate sections before making your selection.

☐ Life sciences ☐ Behavioural & social sciences ☒ Ecological, evolutionary & environmental sciences

For a reference copy of the document with all sections, see nature.com/documents/nr-reporting-summary-flat.pdf

Ecological, evolutionary & environmental sciences study design

All studies must disclose on these points even when the disclosure is negative.

Study description	The study creates a forecast of the energy storage needs for the electric passenger vehicle fleet in the EU and analyzes the potential capacity that can be used for stationary storage applications. It is fully based on publicly available resources and the code used in made fully available as well.
Research sample	Various public sources were used to calibrate the model as described in the manuscript.
Sampling strategy	na
Data collection	The data available from reports and other research were downloaded and duly referenced by the authors. They are saved in the publicly available repository in their original form and the regressions and data handling scripts are available to be reproduced as well.
Timing and spatial scale	Europe from 2020 to 2050
Data exclusions	na
Reproducibility	All original and treated data are available in the repository, making the study fully reproducible.
Randomization	na
Blinding	na

Did the study involve field work? ☐ Yes ☒ No

Reporting for specific materials, systems and methods

We require information from authors about some types of materials, experimental systems and methods used in many studies. Here, indicate whether each material, system or method listed is relevant to your study. If you are not sure if a list item applies to your research, read the appropriate section before selecting a response.

Materials & experimental systems

n/a	Involved in the study
☒	☐ Antibodies
☒	☐ Eukaryotic cell lines
☒	☐ Palaeontology and archaeology
☒	☐ Animals and other organisms
☒	☐ Clinical data
☒	☐ Dual use research of concern
☒	☐ Plants

Methods

n/a	Involved in the study
☒	☐ ChIP-seq
☒	☐ Flow cytometry
☒	☐ MRI-based neuroimaging

Plants

Seed stocks	na
Novel plant genotypes	na
Authentication	na
